# Epidemiological and clinical characteristics of *Streptococcus tigurinus* endocarditis

**DOI:** 10.1186/s12879-019-3914-6

**Published:** 2019-03-29

**Authors:** María Ercibengoa, Miguel Angel Goenaga, Carmen Ardanuy, Immaculada Grau, Cristina García-de-la-Maria, Manuel Almela, Jose María Miro, Enrique Navas, María Carmen Fariñas, Carlos Ruiz de Alegría, Javier de la Torre, Fernando Fernández, Mercedes Marín, Patricia Muñoz, Beatriz Orden, José Antonio Oteo, Lara García-Álvarez, Arístides de Alarcón, José Antonio Lepe Jiménez, Jose María Marimón

**Affiliations:** 1 CIBER Enfermedades Respiratorias-CIBERES, Madrid, Spain; 2 Servicio de Enfermedades Infecciosas, Hospital Universitario, Donostia, Spain; 3 Servicio de Microbiologia, Hospital Universitari de Bellvitge. Institut d’Investigació Biomèdica de Bellvitge (IDIBELL), University of Barcelona, Barcelona, Spain; 4 Servicio de Enfermedades Infecciosas, Hospital Universitari de Bellvitge. Institut d’ Investigació Biomèdica de Bellvitge (IDIBELL), University of Barcelona, Barcelona, Spain; 5 Servicio de Enfermedades Infecciosas, Hospital Clínic de Barcelona, Institut d’ Investigacions Biomèdiques August Pi i Sunyer (IDIBAPS), University of Barcelona, Barcelona, Spain; 6 Servicio de Microbiología, Hospital Clínic de Barcelona, Institut d’ Investigacions Biomèdiques August Pi i Sunyer (IDIBAPS), Barcelona, Spain; 7 Servicio de Enfermedades Infecciosas, Hospital Universitario Ramón y Cajal, Madrid, Spain; 8 Servicio de Enfermedades Infecciosas, Hospital Universitario Marqués de Valdecilla, Universidad de Cantabria, Santander, Spain; 9 Servicio de Microbiología, Hospital Universitario Marqués de Valdecilla, Universidad de Cantabria, Santander, Spain; 10 Grupo de Enfermedades Infecciosas de la Unidad de Medicina Interna, Hospital Costa del Sol, Marbella, Málaga, Spain; 11 Servicio de Microbiología, Hospital Costa del Sol, Marbella, Málaga, Spain; 12 Departamento de Medicina, Servicio de Microbiología y Enfermedades Infecciosas, Hospital General Universitario Gregorio Marañón, Facultad de Medicina, Universidad Complutense de Madrid, Instituto de Investigación Sanitaria Gregorio, Marañón, Madrid, Spain; 13 Departamento de Microbiología, Hospital Universitario Puerta de Hierro Majadahonda, Madrid, Spain; 14 Departamento de Enfermedades Infecciosas, Hospital Universitario San Pedro, Logroño, La Rioja Spain; 15 Clinical Unit of Infectious Diseases, Microbiology and Preventive Medicine. Infectious Diseases Research Group, Institute of Biomedicine of Seville (IBIS), University of Seville/CSIC/University Virgen del Rocío and Virgen Macarena, Sevilla, Spain; 16 Servicio de Microbiología, Hospital Universitario Donostia-IIS Biodonostia, Paseo Dr Beguiristain s/n, 20014 San Sebastián, Spain

**Keywords:** *Streptococcus Viridans*, Infective endocarditis, Multicentre studies, Epidemiologic surveillance, Antibiotic resistance

## Abstract

**Background:**

*Streptococcus tigurinus* was recently described as a new streptococcal species within the viridans group streptococci (VGS). The objectives of the present work were to analyse the clinical and microbiological characteristics of *S. tigurinus* isolated from patients with bacteraemias, to determine the prevalence of *S. tigurinus* among VGS endocarditis in Spain, and to compare the clinical characteristics and outcomes of endocarditis caused by *S. tigurinus* and other VGS.

**Methods:**

Retrospective nationwide study, performed between 2008 and 2016 in 9 Spanish hospitals from 7 different provinces comprising 237 cases of infective endocarditis. Streptococcal isolates were identified by sequencing fragments of their 16S rRNA, *sodA* and *groEL* genes. Clinical data of patients with streptococcal endocarditis were prospectively collected according to a pre-established protocol.

**Results:**

Patients with endocarditis represented 7/9 (77.8%) and 26/86 (30.2%) of the bacteraemias caused by *S. tigurinus* and other VGS, respectively (*p* < 0.001), in two of the hospital participants. Among patients with streptococcal endocarditis, 12 different *Streptococcus* species were recognized being *S. oralis*, *S. tigurinus* and *S. mitis* the three more common. No relevant statistical differences were observed in the clinical characteristics and outcomes of endocarditis caused by the different VGS species.

**Conclusions:**

In this multicenter study performed in Spain, *S. tigurinus* showed a higher predilection for the endocardial endothelium as compared to other VGS. However, clinical characteristics and outcomes of endocarditis caused by *S. tigurinus* did not significantly differ from endocarditis caused by other oral streptococci.

## Background

*Streptococcus tigurinus* was firstly described as a new streptococcal species in 2012 based on DNA hybridization analysis and 16S rRNA sequencing, being genetically very similar to *Streptococcus oralis* [1]. In fact, in 2016, and based on whole-genome sequencing, it was proposed to classify *S. tigurinus* as a *Streptococcus oralis* subspecies [2]. *S. tigurinus*, as other mitis group streptococci has been found as a commensal of the human oral cavity although since its initial description it has been also documented as a causative agent of infective endocarditis (IE) and other infections as meningitis, spondylodiscitis, osteomyelitis, prosthetic infections, etc. [1, 3–7]. The objective of the present study was to establish the clinical and microbiological characteristics of *S. tigurinus* isolated from patients with bacteraemias, to determine the prevalence of *S. tigurinus* among the cases of viridans group streptococci (VGS) endocarditis in Spain, and to compare the outcomes of IE caused by *S. tigurinus* and by other VGS.

## Methods

### *S. tigurinus* prevalence study

To study the relevance of finding *S. tigurinus* in a blood culture, all VGS isolated from blood cultures collected between 2008 and 2016 in two hospitals from Barcelona and San Sebastian, two cities from the north of Spain separated more than 500 km apart, were classified by phenotypic methods and all *S. oralis* were further identified by gene sequencing.

### *S. tigurinus* endocarditis study

To analyse the characteristics of IE caused by *S. tigurinus*, a retrospective nationwide study, performed between 2008 and 2016 in 9 Spanish hospitals located in 7 different provinces comprising 237 cases of IE was performed. Diagnosis of IE was done according to the revised Duke diagnostic criteria [8]. Clinical IE data were prospectively collected according to a pre-established protocol [9]. Only cases with definite IE diagnosis were included in the study.

### Microbiological techniques

VGS isolates were identified by sequencing fragments of their 16S rRNA, *sodA* and *groEL* genes and comparing them with those available at the NCBI and LeBibi databases [10]. A similitude of > 99% with the 3 genes was considered for a correct species identification. *All S. tigurinus* detected were genotyped by MLST according to the established protocol at the Oral Streptococcus MLST Database web page (https://pubmlst.org/oralstrep). Antimicrobial MICs were determined by the broth microdilution method using Iso-Sensitest Broth (Oxoid) supplemented with lysed horse blood 5% *v*/v. After incubation for 24 h at 35 °C, susceptibility results were read and interpreted according to CLSI guidelines. *S. pneumoniae* ATCC 49619 was used as control.

### Statistical analysis

The unpaired t-test or the chi-square test (Fisher’s exact test when appropriate) was used to compare continuous and categorical variables, respectively. All statistical analyses were performed using the online available *GraphPad software (*www.graphpad.com/quickcalcs/*).*

### Ethical considerations

The project and the common case report form were approved by the national and local institutional review boards and ethics committees (E.C. 18/07) and all patients gave their written informed consent to participate in the study.

## Results

In the present work, 169 VGS isolates were identified by molecular methods: 95 from the prevalence study and 74 from the study of IE. The 16S rRNA gene correctly identified all of them, *sodA* misidentified one *S. oralis* isolate as *S. tigurinus* and *groEL* misidentified 5 *S. oralis* isolates as *S. tigurinus* (*n* = 2), *S. cristatus* (n = 2) and *S. mitis* (*n* = 1).

### *S. tigurinus* prevalence study

Overall, 95 cases of VGS bacteraemias recorded in the hospitals of Barcelona and San Sebastian were studied, being 9 identified as *S. tigurinus*. Patients with endocarditis represented 7/9 (77.8%) of the *S. tigurinus* and 26/86 (30.2%) of the remaining VGS (*p* = 0.008). This data suggests a bigger attraction of *S. tigurinus* for the endocardial endothelium as compared with related species of the VGS, a finding that has been also observed in other studies [6].

### *S. tigurinus* endocarditis study

Of the 237 IE caused by VGS recorded in the study, 74 isolates were available for further studies and were identified by phenotypic methods and by gene sequencing. In global, 12 different *Streptococcus* species were recognized being *S. oralis*, *S. tigurinus* and *S. mitis* the three more common causing 37.8, 23.0 and 21.6% of IE cases respectively (Table 1). All *S. tigurinus* had been previously identified by phenotypic methods as *S. oralis* and were found in 5/9 hospitals and in 4/7 provinces. All *S. tigurinus* were fully susceptible to oral penicillin, amoxicillin, and cefotaxime except one isolate that had a penicillin MIC = 0.12 mg/L. Three isolates were tetracycline-resistant (MIC> 4 mg/L) and another three erythromycin-resistant (MIC = 2 mg/L). All *S. tigurinus* isolates were susceptible to clindamycin, levofloxacin and vancomycin. A large heterogeneity of *S. tigurinus* was observed by MLST, having all different ST. Among the 17 *S. tigurinus* isolates, there were only two ST previously defined in the MLST database, ST30 and ST65, both previously identified as *S. oralis* from patients with gingivitis. Comparing IE caused by *S. tigurinus* and other VGS, patients´ average age was higher for *S. tigurinus* endocarditis, without statistical significance (*p* = 0.179 compared to *S. mitis*) (Table 2). No relevant statistical differences were observed in the clinical characteristics of IE caused by the different VGS species. Left heart valves were more frequently affected in *S. tigurinus* IE as well as in other streptococci: 45.9% mitral, 31.1% aortic, and 13.6% both valves. Considering all VGS IE, perforation was the most common intracardiac complication (16.2%), followed by abscess (10.8%) and pseudoaneurysm alone (2.7%) or with perforation (2.7%). In-hospital overall mortality in VGS IE (10.8%) was relatively high as compared with other studies [11]. Mortality due to *S. tigurinus* was quite similar to that of *S. oralis* (11.3% vs 14.8%, *p* = 1). Surprisingly, no mortality was recorded for any of the 16 patients with *S. mitis* IE that was also the group with less patients requiring surgical treatment. Of the patients with IE caused by VGS, 22 were treated only with betalactams (20 with ceftriaxone, 1 with ceftriaxone and ampicillin and 1 with imipenem), 26 with betalactams and gentamicin, being ceftriaxone-gentamicin the combination most frequently used (in 10 patients ceftriaxone-gentamicin alone and in another 8 with a third antibiotic). There were no differences in the antibiotic treatment despite the species identified.

**Table 1:** Species of viridans group streptococci causing infective endocarditis in Spain, 2008–2016

Species	n	%
*S. oralis*	28	37.8%
*S. tigurinus*	17	23.0%
*S. mitis*	16	21.6%
*S. parasanguinis*	3	4.1%
*S. sanguinis*	2	2.7%
*S. pneumoniae*	2	2.7%
*S. infantis*	1	1.4%
*S. salivarius*	1	1.4%
*S. infantarius*	1	1.4%
*S. gordonii*	1	1.4%
*S. anginosus*	1	1.4%
*S. alactolyticus*	1	1.4%
Total	74

**Table 2:** Clinical data of patients with infective endocarditis caused by *S. mitis*, *S. oralis*, *S*. *tigurinus* and other viridans group streptococci, Spain, 2008–2016

	*S. mitis* (*n* = 16)	*S. oralis* (*n* = 28)	*S. tigurinus* (*n* = 17)	Other VGS (*n* = 13)
Demographics				
Age in years: average ± SD (range)	58.4 ± 17.5 (35–94)	60.0 ± 16.7 (32–86)	66.7 ± 17.2 (19–87)	61.2 ± 13.9 (28–80)
Female/Male	2/14	4/24	6/11	5/8
Underlying conditions				
Diabetes mellitus	2	2	0	0
Renal insufficiency	0	1	2	1
Pulmonary disease	3	2	3	1
Neoplasm	2	2	3	1
HIV infection	0	3	0	1
Risk factors				
Previous infective endocarditis	2	5	2	1
Heart failure	1	4	4	2
Atrial fibrillation	3	4	2	1
Site of acquisition				
Nosocomial	0	0	0	0
Community -acquired	15	27	16	12
Health care-related	1	1	1	1
Symptoms at admission				
Affected valve				
Aortic	6	14	7	7
Mitral	6	9	6	2
Aortic + mitral	2	3	2	2
Tricuspid	1	0	0	0
Tricuspid + mitral	0	0	0	1
Aortic + tricuspid + mitral	0	1	0	0
Pulmonary	1	0	0	0
Ductus arteriosus	0	0	0	1
Not determined	0	1	2	0
Presentation				
Fever> 38 °C	16	23	13/16^a^	11
Splinter hemorrhages	1	0	0	2
Osler nodes	0	0	0	1
Janeway lesions	1	0	0	1
New murmur	3	15	5/16	8
Worsening of old murmur	0	4/24	1/15	0
Protein C reactive: average ± SD (range)	74.2 ± 41.5 (13–168)	55.7 ± 45.6 (1–138)	52.3 ± 96.4 (3–356)	40.8 ± 42.7 (3–101)
Elevated Rheumatoid factor	0/2	4/11	1/4	2/4
Vegetations				
Not found	6	6	6	4
Aortic	4	10	6	5
Mitral	3	7	3	1
Tricuspid	1	0	0	0
Aortic + mitral	1	5	2	2
Mitral + tricuspid	0	0	0	1
Chordae tendinae	1	0	0	0
Intracardiac complications				
Perforation	2	4	3	3
Abscess	1	4	1	2
Pseudoaneurysm	0	1	0	1
Pseudoaneurysm & perforation	0	2	0	0
NC	0	1	0	2
Clinical course				
Embolism	1	6/27^a^	5/16^a^	4
New heart failure	1	12/27^a^	5	4
Persistent bacteraemia	1	0	0	1
Surgery				
Indicated	5	19/27^a^	9	7
Performed	5	18/27^a^	6	6
Criteria for surgery^b^				
Cardiac insufficiency	2	9	2	3
Early prosthetic IE	0	1	0	0
Late prosthetic IE	1	2	0	0
Valve insufficiency	3	10	3	4
Embolisms	0	1	0	1
Others	1	5	3	1
Outcome				
Days hospital stay: average ± SD (range)	32.4 ± 19.3 (9–85)	31.0 ± 21.8 (3–106)	29.3 ± 17.9 (5–74)	29.9 ± 16.3 (6–53)
In- hospital mortality	0	4	2	2
1-year mortality^c^	0/14	3/22	2/15	1/8
Recurrence	0	2	2	0
Antibiotic treatment				
Beta-lactams alone	6	9	6	5
Beta-lactams + gentamicin	8	12	7	6
Other combinations	2	7	4	2

^a^Denominator adjusted to patients with data available

^b^Some patients had more than one criteria for surgery

^c^Excluding patents died at hospital

## Discussion

Despite the advances in imaging (echocardiography and nuclear medicine), molecular microbiology and surgery, IE is still today a serious disease with high morbidity and mortality rates. In high-income countries, epidemiology of IE is changing with an increase of elderly patients with prosthetic valves or implantable cardiovascular devices [11]. Also, etiologic agents causing IE seems to be changing, with an increase of staphylococcal IE and a reduction of IE caused by VGS [11, 12]. In this Spanish multicenter study, it was previously shown that VGS represented 27.5% cases of definitive IE [13]. In the present work it has been shown that *S. tigurinus* was responsible for 20% of these definitive IE cases caused by VGS.

*S. tigurinus* has been associated to IE since its first description, although it has been also described as causing meningitis, spondylodiscitis, prosthetic infections, osteomyelitis, and periodontitis among others. However, in a recent review, IE was the most commonly reported manifestation of *S. tigurinus* infection [6] probably because IE has been more systematically searched for than other kind of infections. In that review, no deaths were documented among patients with *S. tigurinus* infection except for one case of osteomyelitis. In our study, 2 patients with *S. tigurinus* IE died during admission with no difference in mortality rates to IE caused by other VGS.

Besides to the oral origin of *S. tigurinus* infections, an enteric source has been also postulated after the translocation of the pathogen from an intraabdominal disorder [14]. However we considered that in most of our patients the origin was the oral cavity as no intraabdominal condition was found in any of the patients, whether the causative agent of the IE was *S. tigurinus* or another of the VSG commonly found in the oral mucosa. The oral origin of most VGS IE highlights the need of an exquisite dental care in patients with risk for IE [15].

IE caused by *S. tigurinus* did not clinically differ from IE caused by other VGS, showing a community-acquired origin, clinical course and outcomes in general better than bacterial IE caused by other Gram-positive bacteria as *Staphylococcus aureus* or *Enterococcus* [11]. Despite the wide genomic heterogeneity most isolates were fully susceptible to commonly used antibiotics in the treatment of IE. An endocarditis should always be suspected when a *S. tigurinus* is isolated from a blood culture due to the high prevalence of IE caused by this, otherwise commensal bacteria.

## Conclusion

In this multicenter study performed in Spain, *S. tigurinus* was a common cause of IE. Clinical characteristics and outcomes of *S. tigurinus* endocarditis did not differ from endocarditis caused by other VGS. *S. tigurinus* showed a high genomic heterogeneity with most isolates susceptible in vitro to antibiotics commonly used in the treatment of IE.

## Abbreviations

CLSI: *Clinical and laboratory standards Institute*
IE: Infective endocarditis
MIC: Minimum inhibitory concentration
MLST: Multi-locus sequence typing
*ST*: *Sequence-type*
VGS: Viridans group streptococci
